# Concussion in Female Athletes of Contact Sports: A Scoping Review

**DOI:** 10.1177/23259671241276447

**Published:** 2024-10-16

**Authors:** Jana Fahr, Oliver Kraff, Cornelius Deuschl, Richard Dodel

**Affiliations:** * Department of Geriatric Medicine, University Duisburg-Essen, Essen, Germany; † Department of Trauma Surgery, Hand and Reconstructive Surgery, University Hospital Essen, University Duisburg-Essen, Essen, Germany; ‡ Erwin L. Hahn Institute for Magnetic Resonance Imaging (ELH), University Duisburg Essen, Essen, Germany; § Institute of Radiology, University Hospital Essen, University Duisburg-Essen, Germany; Investigation performed at the University Hospital Essen, University Duisburg-Essen, Essen, Germany

**Keywords:** concussion, chronic traumatic encephalopathy, fluid-based biomarkers, imaging-based biomarkers, female sex, contact sports

## Abstract

**Background:**

Recent studies have described higher incidences of concussion, with more severe symptoms and worse outcomes in female athletes compared with male athletes.

**Purpose:**

To compile current knowledge about sex-specific differences in incidence, biomechanics, biomarkers, imaging, and outcomes of concussion in athletes participating in contact sports to better understand which fields should be explored in more detail.

**Study Design:**

Scoping review; Level of evidence, 3.

**Methods:**

The PubMed database was searched for articles published between January 2000 and November 2020 using the Medical Subject Headings terms “craniocerebral trauma” and “brain concussion” combined with the contact sports “football,”“soccer,”“hockey,” and “boxing.” Eligibility criteria were based on the recommendations of the Scottish Intercollegiate Guidelines Network. It focused on sex-specific differences within 5 major topics: (1) epidemiology, (2) biomechanics, (3) biomarkers, (4) imaging, and (5) specific concussion outcome variables, including neurocognitive performance, injury severity, and behavioral and psychological symptoms.

**Results:**

A total of 22 studies were included. Eight studies investigated the incidence of concussion, with 4 of the 8 finding a significantly higher incidence rate for female versus male athletes. Six studies that focused on biomechanics found that female athletes received fewer impacts with lower magnitudes. One study addressed biomarkers, showing that S100 calcium-binding protein B and neuron-specific enolase were increased after a game in female athletes, and the level of increase was similar to the changes found in male athletes. Based on the 3 imaging studies, affected brain tissue was greatest in areas associated with tau pathology in chronic traumatic encephalopathy. One study showed a lower hypointensity burden index after a season of ice hockey for female athletes, while another study showed more regions with white matter alterations. Seven studies examined concussion outcomes, with 4 studies showing more severe neuropsychological deficits; in addition, female athletes reported more and worse symptoms than male athletes.

**Conclusion:**

The results of this review indicated that female athletes had a higher risk of sustaining a concussion, although they received fewer impacts with lower magnitudes than male athletes. Biomarkers were able to be used equally for both sexes. Female athletes also had a higher neuropsychological deficit and increasingly worse symptoms after a concussion.

The 2017 consensus statement by the Concussion in Sport Group defines sport-related concussion as a traumatic brain injury (TBI) induced by biomechanical forces.^36^ It may be caused by a direct or indirect impact on the head and can result in neurological symptoms and neuropathological changes. The symptomatic complex of concussion can include a variety of diagnostic signs: somatic, cognitive, and/or emotional symptoms; physical signs; balance impairment; behavior changes; cognitive impairment; and disturbances in the sleep/wake cycle.

Chronic traumatic encephalopathy (CTE) is a feared consequence of repetitive concussions and subconcussive head blows.^39^ It is a neurodegenerative disease in which tau protein accumulates in a unique pattern in neurons and astrocytes, and it tends to be found in athletes of contact sports, military servicemembers, and individuals who have experienced repetitive head traumas. CTE can result in a variety of symptoms and signs, but 2 subtypes exist: 1 with mood and/or behavior features and 1 involving cognitive deficits. In addition, individuals can develop motor impairments. CTE can be diagnosed only on postmortem examination.^38^ Studies on CTE indicate a high likelihood on causality to repetitive head trauma. Since the early 20th century, there have been numerous reports of CTE in former male athletes, but to date, there are no detailed studies focusing on female athletes.

The broad definition and different symptomatic constellations of concussion complicate the diagnosis.^36^ Furthermore, there is no universally valid statement regarding the impact of confounders such as age or sex on the clinical presentation of concussed athletes. In particular, research on concussion focusing solely on female athletes is underrepresented. The results for studies on contact sports–related TBI is nearly 3 times as high without a filter for female sex (1089 vs 368 results in our PubMed search). Most of the results with the filter for female sex had to be excluded because despite the filter, the articles do not include female athletes or focus on sex as a factor.

In Germany, 16% of all players of contact sports are female.^13^ This ranges from 10% in ice hockey to up to 44% in field hockey (Table 1). Recent studies have described higher incidences of concussion^1,6,8,14,18,35^ with more severe symptoms^7,10,35,58^ and worse outcomes^7,10,16,17,35^ in female athletes. The reasons for these differences have been attributed to sex-related differences in the biochemical pathways of the brain: women have a higher resting state cerebral blood flow than men, which may be a compensatory mechanism for the lower blood viscosity due to menstruation, and women show considerable differences in glucose metabolism, inflammatory responses, and connectivity of the brain compared with men. However, knowledge is still missing regarding which biochemical pathways are responsible for the resulting brain tissue injuries after a concussion.

**Table 1:** Number of Female and Male Players of Contact Sports in Germany as of January 2019^13^

Sport	Female Players, n (%)	Male Players, n (%)	Total, n	Clubs, n
American football	23,262 (34.57)	44,033 (65.43)	67,295	450
Boxing	15,416 (19.54)	63,481 (80.46)	78,897	886
Field hockey	38,071 (44.29)	47,879 (55.71)	85,950	376
Ice hockey	2019 (9.8)	18,576 (90.19)	20,595	100
Rugby	3105 (19.13)	13,129 (80.87)	16,234	133
Soccer	1,115,785 (15.64)	6,016,151 (84.36)	7,131,936	25,544
Total	1,197,658 (16.18)	6,203,249 (83.82)	7,400,907	27,489

Another explanation is based on sex-specific biomechanics: Women exhibit lower head-segment mass and neck girth than men, resulting in less isometric neck-muscle strength and neck stiffness.^56^ When a force is applied, women show more angular displacement and acceleration of the head-neck segment despite using more muscle activity area with a faster muscle onset latency.^57^ In addition, poor neck strength has been proposed as a risk factor for concussion.^8^ One study also described a higher intention to report a concussion among female athletes than male athletes.^31^ This could be associated with less conformity to traditional masculine norms and peer pressure from coaches and teammates.

In this review, we aimed to compile the current knowledge about sex-specific differences in the incidence, biomechanics, biomarkers, imaging, and outcomes of concussion in athletes participating in contact sports to better understand which fields should be explored in more detail. The research question driving this study was as follows: To what extent do female athletes experience concussion in contact sports, and are they more prone to long-term postconcussion effects than male players?

## Methods

### Search Strategy and Study Inclusion

The focus of this review was on 5 topics related to concussion: (1) epidemiology, (2) biomechanics, (3) biomarkers, (4) imaging, and (5) specific outcomes, including neurocognitive performance, injury severity, and behavioral and psychological symptoms. Following the PRISMA (Preferred Reporting Items for Systematic Reviews and Meta-Analyses) 2020 guidelines, we searched the PubMed database in November 2020 using the Medical Subject Headings terms “brain concussion” (including the entry terms *brain concussion*, *commotio cerebri*, *cerebral concussion*, *mild concussion*, *intermediate concussion*, *severe concussion*, and *mild traumatic brain injury*) or “craniocerebral trauma” (including the entry terms *craniocerebral trauma*, *craniocerebral injury*, *head trauma*, *head injury*, *crushing skull injury*, *minor head injury*, *multiple head injuries*, *open head injury*, *superficial head injury*, *parietal region trauma*, *occipital region trauma*, *temporal region trauma*, *frontal region trauma*, and *forehead trauma*) combined with “football” (including the entry terms *football* and *rugby*), “soccer,”“hockey” (including the entry terms *ice hockey* and *field hockey*), and “boxing.” The search included articles written in English and published between January 2000 and November 2020, with filters for female sex, adults (age range, 19-44 years), and human participants.

Studies were included if (1) the population included brain concussion or any form of brain trauma, (2) the participants were engaged in contact sports, (3) patients were of female sex and adult age; and (4) ≥1 group of healthy controls or a reference group was included. Studies were excluded if they did not focus on female sex or included children (age, <18 years) and if they were not available as full-text articles in English.

**Figure 1.**
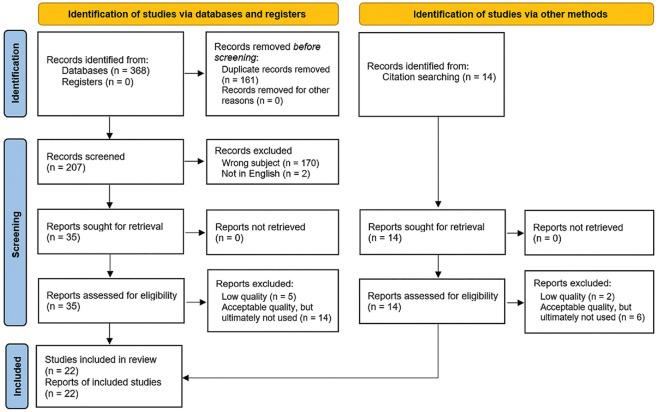
PRISMA (Preferred Reporting Items for Systematic Reviews and Meta-Analyses) 2020 flowchart illustrating search strategy.^41^

A total of 368 articles were initially identified; this was reduced to 207 articles after duplicates were removed. After screening titles/abstracts and excluding articles that did not address our objective (eg, wrong age class, no particular mentioning of female athletes, control group consisting of only male athletes, focus on different sport activities), 35 articles were chosen for further analysis. Additionally, 14 more articles were selected after screening the reference lists of the final chosen articles. The full text of these 49 studies was assessed, and 7 studies were excluded because of their quality; 20 studies were of acceptable quality but ultimately not included in this review or had only an explanatory part (no clinical assessment regarding the focus of this review [sex-based differences in reporting concussion]). Finally, 22 studies^¶^ were included. A flowchart of the study-selection process is shown in Figure 1.

### Quality Assessment

The articles chosen for eligibility were methodologically assessed by 2 authors (J.F., R.D.) independently following the recommendations of the Scottish Intercollegiate Guidelines Network (SIGN)^47^ and the suggestions on scoping reviews as outlined by Peters et al.^43^ Conflicts in the extracted data were discussed between the 2 reviewers until consensus was reached. We used an algorithm by SIGN^48^ to classify the study designs and see which checklist to use (cohort,^49^ case-control,^50^ systematic review/meta-analysis,^51^ or controlled clinical trial^52^).

Key checkpoints for a well-conducted cohort or case-control study according to the SIGN guidelines include a clearly focused question, the selection of patients (comparable groups and participation/dropout rates), the assessment of exposure (reliable methods, blinding), confounding, and statistical analysis (confidence intervals). Controlled clinical trials were assessed to address a clearly focused question, similar groups, a valid measurement of outcomes, and intention-to-treat analysis.

### Outcomes Assessed

#### Epidemiology

Concussion incidence was indicated by injuries diagnosed as concussions per 1000 player-hours (PHs), injuries diagnosed as concussions per 1000 athlete-exposures (AEs), or injury proportion ratios to describe the proportion of injuries diagnosed as concussions.

#### Biomechanics

Biomechanical variables concerning concussion included neck strength as an anthropometric measurement and the frequency and magnitude of impacts received, quantified as linear and rotational head acceleration.

#### Biomarkers

Biomarkers were used to indicate physiological or pathological processes. There have been multiple studies on biomarkers to identify and objectify concussion and CTE, showing that an increase in tau protein,^#^ neurofilament light,^24,40,42,53,63^ S100 calcium-binding protein B (S100B),^12,53^ or neuron-specific enolase (NSE)^53^ appeared to be associated with it. The severity of TBI seemed to correspond with biomarker concentration.^59^ However, these studies consisted solely of male participants or did not investigate sex-based differences.

#### Imaging

While computed tomography is the imaging modality of choice for most acute injuries, it falls short regarding detecting secondary effects such as edema, infarction, and hemorrhage because of its poor soft tissue contrast.^11^ Magnetic resonance imaging (MRI), on the other hand, offers a high soft tissue contrast and a broad range of pulse sequences with different image weightings. Diffusion tensor imaging (DTI) is a noninvasive MRI technique that allows quantitative assessment of brain microstructures. The most common DTI metric is fractional anisotropy (FA) that can be used to describe white matter tract integrity and identify fiber-tract lesions caused by shearing forces in the traumatized brain.^11^ For example, in patients with mild TBI, DTI showed a significant decrease in FA even in the absence of other computed tomography/MRI abnormalities.^30,33^ In patients with moderate to severe TBI, DTI showed related microstructural changes up to ≥18 months postinjury and in correlation with functional outcomes.^62^

#### Concussion Outcomes

The outcome parameters investigated included neurocognitive performance, severity of the injury (measured as time lost before returning to full alertness), and behavioral and psychological symptoms. The symptoms used in different test batteries and the evaluation of their severity are shown in Table 2.

**Table 2:** Symptom Evaluation Scores Used by Included Studies

Symptom Evaluation Score	Symptoms of Concussion	Used by
Sport Concussion Assessment Tool–3 (SCAT 3)^25^	Headache, “pressure in head,” neck pain, nausea or vomiting, dizziness, blurred vision, balance problems, sensitivity to light/noise, feeling slowed down/like “in a fog,”“don’t feel right,” difficulty concentrating/remembering, fatigue or low energy, confusion, drowsiness, trouble falling asleep, more emotional, irritability, sadness, nervousness, or anxiousness	Vedung^58^ (2020)
Immediate Post-Concussion Assessment and Cognitive Testing (ImPACT)^28^	Based on the Post-Concussion Symptom Checklist	Maher^35^ (2014) Covassin^10^ (2013) Stojsih^55^ (2010)
Post-Concussion Symptom Checklist^5^	Headache, nausea, vomiting, balance problems, dizziness, fatigue, trouble falling asleep, sleeping more/less than usual, drowsiness, sensitivity to light/noise, irritability, sadness, nervousness, feeling more emotional, numbness or tingling, feeling slowed down/mentally fogged, difficulty concentrating/remembering, visual problems	Ellemberg^17^ (2007)
Concussion Resolution Index (CRI)^29^	Vomiting, headache, dizziness, nausea, fatigue, weakness, sleep problems, concentration difficulties, memory problems, irritability, depression, nervousness, photophobia, diplopia, sensory abnormalities	Broshek^7^ (2005)

## Results

Of the 22 studies included in this review, there were 8 articles^1,8,14,18,19,34,46,58^ on concussion incidence, 6 articles^6,8,32,55,56,60^ on biomechanics, 1 article^54^ on biomarkers, 3 articles^22,26,44^ on imaging, and 9 articles^**^ on concussion outcomes. Of the included studies, 19 were classified as observational, including 9 cohort,^††^ 4 case-control,^8,17,19,26^ and 6 cross-sectional studies^1,14,18,32,44,56^; 2 studies^16,35^ were classified as systematic reviews/meta-analyses; and 1 study^54^ used a before/after design. A summary of the studies on concussion incidence is shown in Table 3; a summary of the studies on biomechanics, biomarkers, and imaging outcomes is shown in Appendix Table A1; and a summary of the studies on concussion outcomes is shown in Appendix Table A2.

**Table 3:** Characteristics of Studies on Concussion Incidence (n = 8)^*a*^

Lead Author, Year (Country)	Study Design	Sport and Level	Participant Age, y^*b*^	Female, %	Concussion Rate	Results
Agel^1^ 2007 (USA)	Cross-sectional	Ice hockey	College	NR	F: 2.72/1000 PHs M: 1.47/1000 PHs	NR
Collins^8^ 2014 (USA)	Case-control	Soccer, basketball, lacrosse	High school	59.8	F: 4.9/1000 AEs M: 2.5/1000 AEs	OR, 1.8 (95% CI, 1.36-2.49)
Dick^14^ 2007 (USA)	Cross-sectional	Soccer	College	NR	F: 1.42/1000 PHs M: 1.08/1000 PHs	NR
Fraser^18^ 2017 (USA)	Cross-sectional	Basketball, baseball, soccer	College	NR	Ball-contact injuries during study period, n (%) F: 92 (21.2) M: 25 (8.0)	Proportion of ball-contact injuries diagnosed as concussion higher for women than men (IPR, 2.33; 95% CI, 1.63-3.33
Fuller^19^ 2005 (UK)	Case-control	Soccer	Adult	23.7 of PHs	F: 2.6/1000 PHs M: 1.1/1000 PHs	NR
Lopez^34^ 2016 (USA)	Cohort	Rugby	F: 24.3 ± 4.8 M: 23.4 ± 5.0	28.4	F: 8.1/1000 PHs M: 7.6/1000 PHs	No difference (*P* = .593)
Schick^46^ 2003 (Canada)	Cohort	Ice hockey	F: 23.5 M: 20.9	43.7	F: 1.18/1000 AEs M: 0.97/1000 AEs	No difference (*P* = NR)
Vedung^58^ 2020 (Sweden)	Cohort	Soccer	F: 23 ± 4.2 M: 25 ± 4.6	40.6	F: 1.22/1000 PHs M: 1.18/1000 PHs	No difference (*P* = .85)

a AE, athlete-exposure; F, female; IPR, injury proportion ratio; NR, not reported; M, male; OR, odds ratio; PH, player-hour.

b Data are presented as mean or mean ± SD. Competition level is given where specific ages were not provided.

### Concussion Incidence

Of the 8 studies addressing concussion rate, 5 studies^1,14,19,34,58^ reported the rate as injuries per 1000 PHs in matches, and 2 studies^8,46^ reported it as injuries per 1000 AEs in competition and practice. Fraser et al^18^ used the injury proportion ratio. The following sports were investigated: soccer^8,14,18,19,58^ (n = 5), ice hockey^1,46^ (n = 2), and rugby^34^ (n = 1) (see Table 3).

To estimate the injury rate, Agel et al^1^ reviewed data from the National Collegiate Athletic Association (NCAA) Injury Surveillance Program for women's ice hockey. Even though they showed concussions to be the most predominant injury in games and practices, the risk of a player's experiencing a concussion in a game was 8.2-fold higher than in a practice (2.72/1000 AEs vs 0.33/1000 AEs). Compared with another study by Agel et al^2^ on men's ice hockey, women had a higher concussion rate with player contact being the primary mechanism for sustaining a concussion both for male and female athletes. The 2 studies did not directly compare the concussion rates, so there is no report about the statistical significance. Dick et al^14^ used the same methodology as Agel et al^3^ to identify the concussion rate for women's and men's soccer and also observed a higher concussion rate for female athletes. The risk of receiving a concussion was 12-fold higher during a game than during practice.^14^ In a study by Collins et al^8^ concerning neck strength as a risk factor for concussion, data for high school–aged boys’ and girls’ soccer and basketball showed a significantly higher rate of concussions for female athletes. The probability of sustaining a concussion in games was 1.8-fold higher in girls than in boys.

Focusing solely on ball-contact injuries, Fraser et al^18^ used the NCAA Injury Surveillance Program as well, reviewing the data for 11 sport activities. Basketball, baseball, and soccer were comparable between female and male athletes. The other sports activities were single sex (eg, only male football athletes) or could not be compared because of rule differences (eg, no body contact in women's lacrosse). The study showed that women had a significantly larger proportion of ball-contact injuries diagnosed as concussions than men (injury proportion ratio = 2.33; 95% CI, 1.63-3.33).^18^

Fuller et al^19^ studied injury rates using video recordings of FIFA (Fédération Internationale de Football Association) soccer tournaments and found that the concussion rate was 2.4-fold higher for women than for men. The study showed differences in possession status and intent between men and women: Most of the time, injuries in women's soccer resulted from fair challenges and affected the non–ball possessing player, whereas in men's soccer they resulted from unfair challenges with the possessing player's being injured. Most injuries resulted from contact with the upper extremity (35%) and the head (30%). Lopez et al^34^ used the Rugby Injury Survey and Evaluation report tool to catalog concussion injuries in US Rugby 7s tournaments. The difference in concussion rate between female and male players did not reach statistical significance. The major cause for concussions in all players was impact with another opponent in a tackle or collision. Women reported more time loss before returning to full contact play after concussion than men (36.7 vs 27.9 days), a difference that did not reach statistical significance. In contrast, Schick and Meeuwisse^46^ evaluated teams from the Canada West Universities Athletic Association to record injuries. Concussion rates were higher for female than male players, but men had a higher percentage of concussions resulting in more time loss than women. The most common injury region was the head among women and the thigh among men; and 96% of injuries in women, but only 79% in men, resulted from contact with the opponent or the boards. Finally, a study in Swedish elite soccer players showed no significant difference in concussion rate between men's and women's 1st and 2nd Soccer Leagues.^58^

To summarize, of the 8 studies included regarding concussion rate, 7 studies^1,8,14,18,19,34,46^ found a higher concussion rate for women than men, with 2 studies^8,18^ reaching statistical significance (odds ratio = 1.8; 95% CI, 1.36-2.49). The concussion rate was generally higher in games than in practices for both men and women.^1-3,14^ Four studies^1,14,19,46^ showed that the most common mechanism for receiving a concussion was contact with another player.

### Biomechanics

Six articles focused on the biomechanical variables that may increase the risk of sustaining a concussion (see Table A1).^6,8,32,55,56,60^ Brainard et al^6^ equipped female and male ice hockey players with instrumented helmets to quantify the frequency and magnitude of head impacts they received. Female ice hockey players experienced a significantly lower number of impacts per AE (1.7 ± 0.7 vs 2.9 ± 1.2) and significantly lower linear and rotational head acceleration levels than male athletes. Collins et al,^8^ in their study concerning neck strength as a risk factor for concussion, found that concussed athletes had significantly smaller mean neck circumferences, smaller mean ratios of neck circumference to head circumference and lower neck strength (extension, flexion, right and left lateral) than uninjured athletes. They proposed neck strength as a significant predictor for receiving a concussion, reporting that for every 1-lb (0.45-kg) increase in neck strength, the probability of sustaining a concussion decreased by 5%. Tierney et al^56^ found that women exhibited 15% less head-neck segment mass, 5% less head-neck segment length, 12% less neck girth, 50% less isometric neck flexor strength, and 53% less isometric extensor strength than men when heading a ball in a controlled setting. The study also showed that women had 10% greater head acceleration than men.

To measure the frequency and magnitude of direct head impacts in women's soccer, Lamond et al^32^ used smart impact monitor accelerometers and visual on-field monitoring. The authors identified higher mean linear accelerations for unintentional head impacts (head-to-head impacts and unintentional deflections) than purposeful headers (shots, clears, and passes), with the first being responsible for only 4% of all impacts. Stojsih et al^55^ examined male and female amateur boxers and compiled data using Instrumented Boxing Headgear (Simbex), a wireless system comprising 12 single-axis linear accelerometers utilized to measure the translational and rotational accelerations of impacts to the head. The magnitude of impacts was similar for both sexes, but female boxers had significantly lower peak values and had a significantly lower frequency of impacts. Similarly, in a study involving female and male ice hockey players, Wilcox et al^60^ found that female players experienced significantly fewer head impacts (median [interquartile range] for all data) per practice (0.9 [0.6-1.0] vs 1.3 [1.0-1.7]), per game (3.7 [2.5-4.9] vs 6.3 [3.5-9.0]), and per season (169.8 [119.0-230.0] vs 287.0 [201.5-444.6]) (*P* < .001 for all) and sustained impacts that resulted in smaller acceleration magnitudes compared with male players (peak linear acceleration, *g*: 15.0 [14.5-15.5] vs 15.7 [14.8-17.1], *P* = .007; 50th percentile peak rotational acceleration, rad/s^2^: 1211 [1091-1353] vs 1630 [1454-1733], *P* < .001; 95th percentile peak rotational acceleration, rad/s^2^: 3409 [3152-3839] vs 4424 [4076-5182], *P* < .001; HITsp [a composite measure of head impact severity that includes linear and rotational acceleration, impact duration, and impact location]: 13.1 [12.9-13.6] vs 13.6 [13.4-14.1], *P* < .001). In a related study, Wilcox et al^61^ found that male collegiate ice hockey players had greater rotational acceleration during head impacts from contact with another player (mean [95% CI]: 2901.8 rad/s^2^ [2514.5-3348.7 rad/s^2^] vs 2323.0 rad/s^2^ [2031.6-2656.9 rad/s^2^] for female players; *P* = .027) and contact with the boards (3350.4 rad/s^2^ [2995.9-3746.8 rad/s^2^] vs 1859.5 rad/s^2^ [1587.0-2178.8 rad/s^2^] for female players; *P* < .001) and that head impacts occurred more frequently in male versus female players (contact with another player per game: 0.464 vs 0.208; contact with the boards per game: 0.349 vs 0.095; *P* < .001 for both).

The results showed that female athletes experienced fewer head impacts in ice hockey and boxing than male athletes. In female ice hockey players, the impacts were of lower magnitude, with lower linear and rotational accelerations.^6^ In boxing, there was no sex-based difference in the magnitude of impacts.^55^ Concerning anthropometric measurements, studies showed a higher risk of concussion for individuals with smaller neck strength.^8^ In soccer, female players exhibited significantly less neck strength when heading a ball than male players.^56^

### Biomarkers

The study by Stålnacke et al^54^ focused on biomarkers in women, in which venous blood samples were collected from soccer players before and after a competitive game to analyze the levels of S-100B and NSE (see Table A1). For each player, the number of head traumas (headers, collisions, or falls) was estimated using video recordings. The results showed a significant increase in S-100B and NSE after a game (S-100B: 0.18 ± 0.11 vs 0.11 ± 0.05 µg/L [*P* < .001]; NSE: 10.14 ± 1.74 vs 9.05 ± 1.59 µg/L [*P* = .001]). The changes in S-100B were significantly correlated with the number of head traumas (*r* = 0.515; *P* = .001). These results were in accordance with changes observed in studies performed on male athletes, indicating that biomarkers may be used equally for both sexes.

### Imaging

Three articles^22,26,44^ focused on imaging to further understand concussion and brain tissue injuries (see Table A1). Ghajari et al^22^ used injury modeling to simulate different types of head impacts (helmet-to-helmet collisions, falls from ground level, and road traffic accidents) to map the contours of strain and the strain rate at the gray matter–white matter boundary. Using DTI, this was also performed for patients with TBI. The results showed that deformation in the brain tissue was greatest in sulcal locations, which is a typical location for tau pathology in CTE. Both men and women were included in this study, but the results were not differentiated between the sexes. Helmer et al^26^ examined male and female ice hockey players before and after a sports season. They used susceptibility-weighted imaging to identify changes in brain tissue and cerebral microbleeds and calculated a *hypointensity burden index*. Female ice hockey players had a lower hypointensity burden index than male ice hockey players at the beginning and end of the season. The rise in the burden was significant for male ice hockey players but not for female ice hockey players. A study by Rubin et al^44^ on heading in soccer used DTI and FA to determine sex-based differences in cerebral white matter microstructural alterations. In women, they found 8 regions in which greater heading exposure was associated with lower FA; in men, they found only 3. In most regions where women showed the strongest associations between heading and lower FA, the results in men were in the opposite direction.

The 3 imaging-related studies showed that deformation in brain tissue is greatest in the locations that are associated with tau pathology in CTE.^22^ Female ice hockey players had a lower rise in hypointense brain regions after a season than male ice hockey players.^26^ More regions with microstructural white matter alteration after heading exposure could be found in female than in male soccer players.^44^

### Concussion Outcomes

The outcome parameters investigated included neurocognitive performance, severity of the injury (measured as time lost before returning to full alertness), or behavioral and psychological symptoms. A total of 9 articles^§§^ on concussion outcomes were identified in our search (see Table A2). Broshek et al^7^ examined high school and collegiate athletes of different sport activities (soccer, field hockey, lacrosse, basketball, cheerleading, football, wrestling, and other) by using the Concussion Resolution Index (CRI)^29^ at baseline and after a concussion. The CRI is a neurocognitive assessment tool that tests simple and complex reaction time (SRT and CRT, respectively) and processing speed (PS) and uses a checklist for postconcussion symptoms. Female athletes demonstrated a greater decline in cognitive function (significant for SRT and CRT, nonsignificant for PS) and demonstrated significantly more symptoms. The pattern of symptoms was similar between male athletes and female athletes, although women reported significantly more often concentration problems, fatigue, lightheadedness, and visual flyspecks. There were no sex-based differences in concussion severity, but male athletes experienced loss of consciousness nearly twice as often as female athletes.

To detect sex-based differences in neurocognitive performance and symptoms after concussion, Covassin et al^10^ tested concussed soccer players using the Immediate Post-Concussion Assessment and Cognitive Testing (ImPACT) test battery^28^ while also adjusting for body mass index. The authors found significantly worse performances on verbal and visual memory for female athletes at baseline and 8 days after concussion. Additionally, concussed female athletes expressed a greater number of total concussion symptoms (migraine, affective, somatic, or sleep disturbances) and reported more symptoms on the migraine-cognitive-fatigue and sleep clusters 8 days after concussion than male athletes. There were no sex-based differences regarding motor PS or reaction time.

Dougan et al^16^ conducted a large meta-analysis including 78 studies to determine which preexisting athlete characteristics were associated with greater decline in neurocognitive functioning 10 days after concussion. The sports included football (both Australian Rules and American), boxing, ice hockey, rugby, and soccer. Along with younger age, high school level of competition, and fewer years of education, the authors found that female sex was a key risk factor for greater deficits in neuropsychological function, although male athletes reported more symptoms. The analysis did not report the test batteries or symptom complexes used by the included articles, and the included studies on American or Australian Rules football had recruited only male athletes.

A study on prolonged cognitive outcome on solely female soccer players was conducted by Ellemberg et al,^17^ who used the Post-Concussion Symptom Checklist^5^ and 10 neuropsychological tests 6 to 8 months after a concussion to assess executive function, attention, visuomotor skills, learning, and memory. Compared with a control group of nonconcussed athletes, the cases showed a significant decrease in inhibition speed, flexibility, planning time, and response time.

In their study concerning concussive injuries in rugby, Lopez et al^34^ found that women needed significantly more days to return to full-contact play than men (36.7 vs 27.9 days). In contrast, Schick and Meeuwisse^46^ conducted a study on injury profiles in ice hockey, showing that women missed fewer exposures after an injury than men (4.15% vs 8.05% of total exposures). This percentage does not focus solely on concussions, although they caused the most time lost from participation both for female and male athletes.

In a 2014 review by Maher et al^35^ that examined concussion and heading in soccer, including neurocognitive outcomes, 1 included study by Colvin et al^9^ described poorer reaction time scores for female athletes and worse performance in the memory component and the visual-processing composite score for previously concussed players. A study by Zuckerman et al^64^ reported no significant differences between the sexes at baseline and within 10 days of injury using the ImPACT^28^ test battery. In both studies,^9,64^ female athletes reported significantly more symptoms than male athletes. Whether a general difference in the type of the reported symptoms between men and women was found was not specified.

Stojsih et al^55^ examined the magnitude and frequency of impacts in male and female amateur boxers. To evaluate the link between punch biomechanics and cognitive function in boxing, they compared data from the Instrumented Boxing Headgear, ImPACT concussion management software,^28^ and 2 scores for injury severity (Head Injury Criterion^21^ and Gadd Severity Index^20^). The scores were similar between male and female boxers, and the study assessed mostly subconcussive blows, with no concussions being detected. The athletes showed a significant decrease in the delayed memory scores, with no sex-based differences and no other impairment of neurocognitive function. Vedung et al^58^ studied Swedish elite football players using the SCAT 3.3^25^ and found worse initial symptoms and a higher total number of symptoms for female athletes at 48 hours and 3 months postconcussion. Women needed a median of 20 days to return to full contact play, whereas men needed a median of 10 days. Players with a previous sport-related concussion had a higher number of baseline symptoms and a higher risk of sustaining another concussion.

To summarize, 4 studies^7,10,16,35^ of 5 showed a higher deficit in neurocognitive performance after a sports-related concussion for female athletes than for male athletes, whereas 1 study^55^ found no difference between the sexes. Four^7,10,35,58^ of the 5 studies focusing on the number and severity of symptoms described female athletes’ reporting of more and worse symptoms, and the opposite was found by the other study.^16^ Two studies found no difference between male and female athletes in terms of injury severity.^7,55^ One study^46^ showed a higher percentage of missed exposures after injury for male athletes (not focusing solely on concussions), while 2 studies^34,58^ described a longer time to return to full contact play for female athletes.

## Discussion

This review identified 22 articles concerning the incidence, biomechanics, biochemicals, imaging, and outcome of concussion in female athletes of contact sports. Female athletes were found to have a significantly higher incidence rate of concussion in games and practices of soccer and ice hockey.^1-3,8,14,18,19^ Further research in football and rugby is needed for a broader statement on concussion incidence in female athletes of contact sports, especially since in both sports contact is needed to win back the ball or gain progress in the game. The most common mechanism for receiving a concussion in ice hockey and rugby was contact with another player, for both male and female players.^1,2,34^ This is surprising, especially for those studies conducted in ice hockey, as the rules state that body checking is illegal for female players but not for male players.

Regarding biomechanical variables, female athletes experienced fewer head impacts in ice hockey and boxing than male athletes.^6,55,60^ Individuals with lower neck strength had a higher risk of receiving a concussion.^8^ In soccer, women were found to exhibit significantly less neck strength when heading a ball than men.^56^ There was no research examining neck strength in sports activities other than soccer or scenarios other than heading a ball.

The study on biomarkers related to brain tissue damage found that female soccer players had a significant increase in S-100B and NSE after a game.^54^ The changes in S-100B correlated with the number of head traumas. This is in accordance with changes observed in studies performed on male athletes indicating that biomarkers may be used equally for both sexes. To further confirm this statement, research to affirm the results as well as studies on the changes in tau and neurofilament light need to be conducted.

The included studies on imaging after concussion found that deformation as visualized by functional cranial MRI in brain tissue was greatest in the locations that are associated with tau pathology in CTE.^22^ Female ice hockey players were found to have a lower hypointensity burden index after a season than male ice hockey players.^26^ Female soccer players also showed more white matter alterations after heading exposure than male soccer players.^44^

Research focusing on concussion outcomes found a higher deficit in neurocognitive performance following a sports-related concussion for female athletes than for male athletes.^7,10,16,17,35^ Women reported more and worse symptoms.^7,10,35,58^ There was a discrepancy regarding the difference in the severity of injuries (measured as the days needed before returning to full-contact play) between male and female athletes. According to a critical literature review by Dick,^15^) evidence exists for sex-based differences in outcomes of non–sports related TBI, in which studies showed a worse outcome for women in 85% of 20 measured variables, which were primarily somatic symptoms, as well as a 1.28-fold higher mortality rate after TBI.

The results of this review indicated that women had a higher risk of sustaining a concussion in contact sports, despite receiving fewer impacts with lower magnitudes, and they had a higher deficit in neurocognitive performance and more and worse symptoms afterward. There have been various hypotheses to explain these sex-based differences:

- Women have a higher resting state cerebral blood flow, which may be a compensatory mechanism for the lower blood viscosity due to menstruation, and show considerable differences in glucose metabolism, inflammatory responses, and connectivity of the brain.^23^
- Women exhibit less neck strength than men.^56^ Poor neck strength is a risk factor for concussion; for every pound increase in neck strength, the probability of sustaining a concussion decreases by 5%.^8^
- Women have a higher intent to report a concussion.^31^ This could be associated with less conformity with traditional masculine norms and peer pressure from coaches and teammates.

### Limitations

Despite an elaborated systematic approach to the research question, there were some limitations to this review. (1) We searched only the PubMed database, which may result in a limited article inclusion. To overcome this, we also searched the references of the included articles for further published research. (2) Furthermore, we excluded children and young persons in our analyses; however, even though this is an important issue, only a few studies were identified where concussion and its results in children were investigated. (3) Important clinical outcomes and the impact on physical health were considered; however, the impact on patient-reported outcomes such as health-related quality of life, social psychological health, social relationships, and others has been neglected.

## Conclusion

There is evidence that female sex is a risk factor for a higher injury rate of concussion and worse outcome (neuropsychological deficits and more and worse symptoms). Female athletes have a higher neuropsychological deficit and increasingly worse symptoms following a concussion. Biomarkers appear to be equally usable for both sexes, but more research is needed to clarify this.

There are existing differences in rules between men's and women's sports, such as forbidden body checking in women's ice hockey, which do not seem to benefit women.^1^ Studies in the future could potentially examine whether more rule adaptations are needed, such as the banning of “stiff-arms” in football or banning of headers in soccer, and if women profit from those adaptations. More research is urgently needed to clarify the differences in concussion between male and female athletes.

## Appendix

**Table A1:** Characteristics of Studies on Concussion Biomechanics, Biomarkers, and Imaging^*a*^

Lead Author, Year (Country)	Study Design	Sport	Participant Age, y^*b*^	Female,%	Methods	Results
Biomechanics (n = 6)						
Brainard^6^ 2012 (USA)	Cohort	Ice hockey	F: 19.9 ± 1.1 M: 21.4 ± 1.4	58	Helmet unit with 6 single-axis linear accelerometers	Impact exposures were less frequent and of lower magnitude for women than men
Collins^8^ 2014 (USA)	Case-control	Soccer, basketball, lacrosse	High school	59.8	Anthropomorphic testing with handheld dynamometers and tension scales	Neck strength was a significant predictor of concussion
Lamond^32^ 2018 (USA)	Cross-sectional	Soccer	F: 19.7 ± 1.2	100	Smart Impact Monitor accelerometers	Nonheader impacts resulted in higher mean linear accelerations than purposeful headers
Stojsih^55^ 2010 (USA)	Cohort	Amateur boxing	F: 24 M: 22	50	Impact boxing headgear	Magnitude of impacts was similar for both sexes. There were significantly fewer impacts in women
Tierney^56^ 2008 (USA)	Cross-sectional	Soccer	F: 19.5 ± 1.8 M: 20.3 ± 2.9	65.9	Anthropometric, isometric strength (dynamometer) and head kinematics (mouthpiece accelerometer) assessments	Lower head-neck segment stability qualities and greater head accelerations in women than men
Wilcox^60^ 2014 (USA)	Cohort	Ice hockey	College	58.6	Head Impact Telemetry System	Lower number of impacts with lower acceleration magnitudes in women than men
Biomarkers (n = 1)						
Stalnacke^54^ 2006 (Sweden)	Before/After	Elite soccer	F: 23 ± 3	100	S-100B, NSE	Concentrations of S100-B and NSE were increased after a game; changes in S100-B correlated with the number of headers/other trauma events
Imaging (n = 3)						
Ghajari^22^ 2017 (UK)	Cohort	Professional American football	Control: 35.52 ± 17.65 TBI: 38.01 ± 12.45	40.5	Injury modeling (helmet-to-helmet collision, fall from ground level/road traffic accident), DTI/FA	Brain tissue deformation induced by head impact loading was greatest in sulcal locations
Helmer^26^ 2014 (Canada)	Case-control	Ice hockey	F: 21 ± 4 M: 23 ± 2	44.4	SWI, hypointensity burden index	Women had a lower HBI than men at the beginning and the end of the season; rise in the burden was significant for men but not for women
Rubin^44^ 2018 (USA)	Cross-sectional	Soccer	F: 25.8 (20-28) M: 25.7 (20-28)	50	DTI, FA	In women more widespread evidence of microstructural white matter alteration than in men

a DTI, diffusion tensor imaging; FA, fractional anisotropy; F, female; HBI, hypointensity burden index; M, male; NR, not reported; NSE, neuron-specific enolase; S100B, S100 calcium-binding protein B; SWI, susceptibility weighted imaging; TBI, traumatic brain injury.

b Data are presented as mean, mean ± SD, or mean (range). Competition level is given where specific ages were not provided.

**Table A2:** Characteristics of Studies on Concussion Outcomes (n = 9)^*a*^

Lead Author, Year (Country)	Study Design	Sport	Participant Age, y^*b*^	Female, %	Methods	Neurocognitive Performance	Symptoms	Injury Severity/Time Loss
Broshek^7^ 2005 (USA)	Cohort	Soccer, field hockey, lacrosse, basketball, cheerleading, football, wrestling, other	F: 17.5 M: 19.7	24.5	CRI	Greater declines in cognitive function in women than men	More symptoms in women; similar constellations of symptoms between sexes	No difference between sexes
Covassin^10^ 2013 (USA)	Cohort	Soccer	F: 17.78 ± 2.3 M: 17.69 ± 2.1	58.9	ImPACT	Lower performance on visual memory composite scores in women than men	Higher scores on total symptoms in women than men	NR
Dougan^16^ 2014 (Australia)	Meta-analysis	Australian Rules football, boxing, American football, ice hockey, rugby, soccer	Adolescent/ adult	NR	Comprehensive Meta-Analysis Version 2	Female sex was a key risk factor for greater neuropsychological deficits 10 days after concussion	Fewer symptoms 10 days after concussion in women than men	NR
Ellemberg^17^ 2007 (Canada)	Case-control	Soccer	F cases: 22.7 F controls: 22.3	100	California Verbal Learning Task, Ruff 2&7 Selective Attention Test, Brief Test of Attention, Symbol Digits Modalities Test, Stroop Color Word Test, Tower of London–Drexel University, Letter Fluency Test, Forward/Backward Digit Span, Simple Reaction Time, Choice Reaction Time	Women experienced prolonged cognitive sequelae after a first sports-related concussion	NR	NR
Lopez^34^ 2016 (USA)	Cohort	Rugby	F: 24.3 ± 4.8 M: 23.4 ± 5.0	28.4	Time loss in days	NR	NR	More days needed to return to full-contact play in women vs men
Maher^35^ 2014 (Canada)	Review	Soccer	Youth/ adolescent/ adult	NR	NR	Worse performance on neuropsychological tests in female players	Significantly more symptoms of concussion in women than men	NR
Schick^46^ 2003 (Canada)	Cohort	Ice hockey	F: 23.5 M: 20.9	43.7	Time loss in days	NR	NR	Fewer sessions missed after injury in women than men
Stojsih^55^ 2010 (USA)	Cohort	Boxing	F: 24 M: 22	50	ImPACT Concussion management software, HIC, GSI	Decrease for delayed memory postscores for both sexes after subconcussive blows, no other neurocognitive impairments, no difference between sexes	NR	No significant difference between sexes
Vedung^58^ 2020 (Sweden)	Cohort	Soccer	F: 23 ± 4.2 M: 25 ± 4.6	40.6	SCAT	NR	Worse initial symptoms and a higher number of symptoms 48 h and 3 mo after concussion in female vs male players	More days needed to return to full contact play for female vs male players

a CRI, Concussion Resolution Index; F, female; GSI, Gadd Severity Index; HIC, Head Injury Criterion; ImPACT, Immediate Post-concussion Assessment and Cognitive Test; M, male; NR, not reported; SCAT, Sport Concussion Assessment Tool.

b Data are presented as mean, mean ± SD, or mean (range). Competition level is given where specific ages were not provided.

## Notes

¶ References 1, 6-8, 10, 14, 16-19, 22, 26, 32, 34, 35, 44, 46, 54-56, 58, 60.

# References 4, 12, 24, 27, 37, 40, 42, 45, 53, 63.

** References 7, 10, 16, 17, 34, 35, 46, 55, 58.

†† References 6, 7, 10, 22, 34, 46, 55, 58, 60.

§§ References 7, 10, 16, 17, 34, 35, 46, 55, 58.
